# Study of PLA pre-treatment, enzymatic and model-compost degradation, and valorization of degradation products to bacterial nanocellulose

**DOI:** 10.1007/s11274-023-03605-4

**Published:** 2023-04-17

**Authors:** Georgia Sourkouni, Sanja Jeremić, Charalampia Kalogirou, Oliver Höfft, Marija Nenadovic, Vukasin Jankovic, Divya Rajasekaran, Pavlos Pandis, Ramesh Padamati, Jasmina Nikodinovic-Runic, Christos Argirusis

**Affiliations:** 1 Clausthal Centre for Materials Technology (CZM), Clausthal University of Technology, Leibnizstr. 9, 38678 Clausthal-Zellerfeld, Germany; 2 Institute of Molecular Genetics and Genetic Engineering (IMGGE), University of Belgrade (UB), Vojvode Stepe 444a,, 11042 Belgrade 152, Serbia; 3 School of Chemical Engineering, National Technical University of Athens, 9 Heroon Polytechneiou St., Zografou Campus, 15773 Athens, Greece; 4 Institute for Electrochemistry, Clausthal University of Technology, 38678 Clausthal-Zellerfeld, Germany; 5 School of Chemistry, Trinity College Dublin, College Green, Dublin 2, Ireland

**Keywords:** PLA, AOPs pre-treatment, Enzymatic degradation, Model-compost degradation, Surface properties modification, By-product valorisation, Bacterial nanocellulose

## Abstract

It is well acknowledged that microplastics are a major environmental problem and that the use of plastics, both petro- and bio- based, should be reduced. Nevertheless, it is also a necessity to reduce the amount of the already spread plastics. These cannot be easily degraded in the nature and accumulate in the food supply chain with major danger for animals and human life. It has been shown in the literature that advanced oxidation processes (AOPs) modify the surface of polylactic acid (PLA) materials in a way that bacteria more efficiently dock on their surface and eventually degrade them. In the present work we investigated the influence of different AOPs (ultrasounds, ultraviolet irradiation, and their combination) on the biodegradability of PLA films treated for different times between 1 and 6 h. The pre-treated samples have been degraded using a home model compost as well as a cocktail of commercial enzymes at mesophilic temperatures (37 °C and 42 °C, respectively). Degradation degree has been measured and degradation products have been identified. Excellent degradation of PLA films has been achieved with enzyme cocktail containing commercial alkaline proteases and lipases of up to 90% weight loss. For the first time, we also report valorization of PLA into bacterial nanocellulose after enzymatic hydrolysis of the samples.

**Supplementary Information:**

The online version contains supplementary material available at 10.1007/s11274-023-03605-4.

## Introduction

Fossil-based polymers are still widely used due to their versatility, light weight, durability, and low production cost, leading to their uncontrolled disposal in the environment. Currently, these polymers represent the most common pollutants, accounting for 54% of all human generated waste material (Hoellein et al. 2014; Venkatesh et al. 2021).

Current methods of managing polymer materials are landfilling, cement clinker co-processing, recycling by matrix degradation (i.e., use of microorganisms, electrolytes, solvents), mechanical recycling through matrix decomposition, high voltage fragmentation and microwaves (Mativenga et al. 2017; Wong et al. 2017). Bioplastics are seen as suitable alternative for a while now, but their participation in plastic market is still below 2% (Mos Moshood et al. 2022; Babu et al. 2013). Bioplastics help pave the way to circular economy of plastic materials, since they are produced from renewable resources and offer more end-of-life options (Jeremic et al. 2020). Landfilling should be avoided in the future as it terminates the opportunities to gain more value through circular thinking (i.e., recycle, reuse, reduce etc.) (Korniejenko et al. 2021; Levanen et al. 2021; Zorpas et al. 2021).

Due to its ability to be biosynthetic and biodegradable, PLA is considered the most promising environmentally friendly material for a sustainable bioeconomy (Xu et al. 2021). Biotechnologically produced PLA is of variable composition in terms of the ratio of L- and D-lactic acid monomers, therefore it has varying physiochemical characteristics and can replace polypropylene and polyethylene in a variety of applications such as food packaging, agriculture, biomedicine, 3D printing (Reeve et al. 1994; Bandopadhyay et al. 2018; DeStefano et al. 2020; Ncube et al. 2020). PLA eliminates the need to use limited petroleum resources for production thus lowering the energy consumption by 25–55% (Arias-Nava et al. 2021). Since its commercialization in 1997, global PLA production reached 525 million US dollars in 2020 and is expected to increase to 1.82 billion US dollars in 2028 (https://www.statista.com^2022^).

Marketing PLA as biodegradable polymer is misleading to consumers as it makes one believe that PLA can be simply degraded by landfilling it. PLA is compostable according to ASTM International D5338-15 standard, at an indicative temperature of 58 ± 5 °C (Sun et al. 2022). This makes PLA recalcitrant under the conditions present in landfills, and degradable only if composting is done industrially at elevated temperatures, which requires developing specialized infrastructure and high energy consumption. This particularly poses a problem for developing countries (Steiner et al. 2022). As the compost is further distributed for agricultural applications, the current PLA degradation strategy contributes to microplastics pollution in the environment that pose an even greater threat as they can concentrate pollutants and are easily spread across all ecological niches (Mato et al. 2001; Rochman 2018). On the other hand, complete degradation of PLA in nature can last even decades, while the degradation rate in seawater is even lower (Nampoothiri et al. 2010; Goswami et al. 2020; Upadhyay et al. 2020).

Both biodegradation and composting rely on depolymerization mediated by microorganisms and their enzymes. PLA degraders are amongst the least studied plastic degrading microorganisms. The depolymerisation of PLA and polymers in general cleaves their chemical backbone, resulting in into intermediate metabolites, such as monomers, dimers, and oligomers (Yasin et al. 2022; Qi et al. 2017). The most represented fungal PLA degraders are Ascomycota and Basidiomycota (Satti et al. 2017), while different families belonging to Actinobacteria showed PLA-degrading ability (Butbunchu and Pathom-Aree 2019; Kawai 2010), with *Amycolatopsis* and *Actinomadura* as the most potent genera (Pranamuda and Tokiwa 1999). Other PLA-degrading bacteria predominantly belong to Bacillaceae*,* in fact, the majority of the PLA-degrading enzymes, classified as alkaline proteases, are secreted from *Bacillus* species (Butbunchu and Pathom-Aree 2019; Oda et al. 2000a; Tokiwa and Calabia 2006). An increasing number of mesophilic PLA degraders are discovered in the last 10 years (Kim and Park 2010; Pattanasuttichonlakul et al. 2018; Decorosi et al. 2019). Recently, Richert and Dabrowska (2021) have tested different compost and activated sludge extracts and compared them with commercial enzymes showing that the first have the greatest impact on the biodegradation of PLA/PCL mixtures and pure PCL, while the commercially available microorganisms degrade PLA more efficiently. Morohoshi et al. (2018) have also shown the importance of the biofilm formation on the surface of biodegradable plastic for their degradation.

The structural homology of PLA with natural polymers permits its degradation by naturally occurring enzymes grouped as PLA depolymerases. According to primary substrate specificities, PLA depolymerases are classified as protease-type and lipase-type, the latter also including cutinases. Both types of PLA depolymerases are sharing serin hydrolase catalytic mechanisms, but significant differences in the stereochemistry of catalytic sites between them give a structural basis for different substrate specificities (Rosato et al. 2022; Kawai et al. 2011). Protease-type PLA depolymerases are strictly specific for PLA containing l-lactate, used in most studies, due to structural homology to proteins composed of L-aminoacids (Reeve et al. 1994). For a long time, it was thought that protease-type PLA depolymerases are more common and more efficient as comparative studies using PLLA as a substrate identified an abundance of commercial proteases, of both mammalian and microbial origin, capable of PLA degradation, as well as a general lack of commercial lipases for that purpose (Hoshino and Isono 2002; Masaki et al. 2005; Lim et al. 2005).

Among factors affecting the enzymatic polymer degradation rate crystallinity plays an important (Cui et al. 2022; Li and McCarthy 1999). The crystalline structure of PLA is an obstacle to enzymatic degradation at ambient conditions and poses a demand for material pretreatment, most commonly thermal pretreatment at temperatures close to PLA glass transition temperature (60 °C) (Karamanlioglu et al. 2017).

Enzymatic adsorption on the PLA surface was studied with Proteinase K as a model system and is an irreversible process in aqueous solutions, as degradation of PLA continues even after the enzyme solution replacement with a buffer (Yamashita et al. 2005; Bose et al. 2015). Amorphous regions of PLA are first subjected to enzymatic hydrolysis, so very often overall crystallinity increases during the initial enzymatic degradation and only later do crystalline regions begin to degrade (Kawai et al. 2011; Lee et al. 2014; Vasanthan and Gezer 2013). UV irradiation reduces the molar mass of PLA, which is known to have a positive effect on degradation rate, and therefore is a suitable alternative to thermal pretreatment (Pattanasuttichonlakul et al. 2018; Tsuji and Miyauchi 2001; Longieras et al. 2007).

Recently, we have shown that the surface chemistry and composition of PLA is improved towards bacterial degradation (Sourkouni et al. 2021; Kalogirou et al. 2022). Several other studies have shown that photodegradation yields positive effects on the biodegradation of polymers by deteriorating the polymer surface and increasing the surface availability for biodegradation (Yang et al. 2005; Longieras et al. 2007; Jeon and Kim 2013; Vimala and Mathew 2016). To enable the efficient docking of microorganisms and enable subsequent polymer degradation, a surface pretreatment may be necessary, especially to enrich surface with oxygen.

One can distinguish two types of polymers pre-treatment to enhance their degradability: thermal and non-thermal pre-treatment methods. It has been reported that not pre-treated PLA did not biodegrade under anaerobic conditions, while pre-treatment influenced the hydrolysis reaction rate, which increased the production of biogas during the biodegradation (Benn and Zitomer 2018; Wei and Zimmermann 2017). The non-thermal pretreatments can be categorized in (i) ultraviolet (UV) irradiation, (ii) high-power ultrasounds, (iii) grinding, (iv) plasma treatments and possible combinations of them (Yasin et al. 2022; Sourkouni et al. 2021; Kalogirou et al. 2022).

The objective of this study was to assess the impact of advanced oxidation processes as pretreatment technologies for PLA biodegradation at ambient to mesophilic conditions and to examine the effect on the enzymatic degradation of PLA materials as this methodology requires less energy and allows products of degradation to be further valorized. Overall, the present work aims to further contribute to a general understanding of the biodegradability of PLA under different environmental conditions which should be of primary importance in order to shift PLA from industrially compostable to fully biodegradable polymer.

## Materials and methods

### PLA films sample preparation

PLA used in this study was received from Nature works, Ingeo (4043D). The crystallinity of the pellet was 35.2%, the melting point 152.3 °C, and MFR, 6 g/10 min. MRF (Mass flow rate) is the measure of polymers flow behaviour. It measures the ease of flow of melted plastic, which is important to decide the pressing condition for making PLA films from pellets. It is expressed in a standard unit of g/10 min.

The melting point of PLA and the crystallinity of the PLA pellets and prepared films were measured based on the DSC analysis of the samples. The melting point of PLA polymer was measured at various times are and was found in the range of 152.3–153.6 °C.

Prior to processing the PLA pellets were dried for 50 °C for 5 h. Polymer pellets were pressed into films (ca. 1 mm thickness) using a Servitech Polystat 200 T compression press at 180 °C. The compression was held at 10 bar for 2 min, followed by 100 bar and 200 bar for 1 min each. The compressed sheet is crash cooled using tap water for 30 s and demoulded. The crystallinity of the prepared film was measured to be 1.9%.

PLA samples pretreated with ultraviolet (UV) waves for 6 h, ultrasonic waves (US) with a frequency of 20 kHz and 860 kHz, as well as the combinations of UV and US treatments, were cut into pieces (1 cm × 2 cm), weighted, rinsed with 70% (v/v) ethanol and air dried. Ethanol was used for disinfection of the sample surface to reduce the possibility of contamination, since it is rapidly bactericidal and evaporates fast at room temperature. This method of sterilizing samples prior to degradation experiments has been used in numerous studies (Syranidou et al. 2019; Mandic et al. 2019) with no significant effect on material performance or the course of the experiment. We certainly treated the control samples in the same way to ensure which changes are the result of the biodegradation itself.

All samples were prepared in duplicates, including non-treated control PLA sample. Details on the sample preparation as well as on the pre-treatment methods can be found in our previous publications (Sourkouni et al. 2021; Kalogirou et al. 2022).

### Biodegradation in model compost

Biodegradation of control and pretreated PLA samples was performed in model compost according to the previously described protocol (Ponjavic et al. 2017a; Tomšič et al. 2022). Experiment was set up in glass Petri dishes (120 mm diameter, 30 mm height), in 150 g of compost per Petri dish. Samples were placed inside the compost at a depth of 1 cm. Petri dishes were incubated at 37 °C for 10 and 24 weeks, and the humidity of the compost was maintained around 50% by weight. At the end of experiment samples were rinsed with 70% (v/v) ethanol, air dried and weighted.

### Microbial cell counts

In order to assess the number of viable microbial cells per gram of model compost before and after biodegradation experiment, colony forming units were determined by following standard protocols (Tomšič et al. 2022; Lee et al. 2021). Compost samples (1 g) were resuspended in distilled water, and serial dillutions prepared and plated on three types of growth media: Luria–Bertani agar (LA, Oxoid, UK) for heterotrophic bacteria; mannitol soy flower agar (MSF, Difco, UK) for sporulating aerobic bacteria and Sabouraud dextrose agar (SAB, Difco, UK) for fungi. Bacterial and fungi counts, determined 24 h and 72–96 h after plating, respectively, were expressed as colony forming units per gram of soil (CFU/g).

### DSC analysis

DSC analysis was performed using PerkinElmer Pyris Diamond Scanning Calorimeter to understand the changes in the crystallinity among the powder, film, and pellet. The samples (3–5 mg), sealed in aluminum pans were used for the measurements. The samples are heated from − 100 to 2000 °C at the rate of 10 °C/min. The samples are crash cooled to − 100 °C to determine the enthalpy of cold crystallization. The percentage of crystallinity was calculated from the DSC data using the equation given below by considering the melting and cold crystallization enthalpy (Table 1).

$$\% Crystallanity=((\Delta Hm-\Delta Hcc)/\Delta H^\circ m)\times 100$$

where *ΔΗ*_m_ = Enthalpy of melting, *ΔH*_cc_ = Enthalpy of cold crystallization, *ΔH*°_m_ = Enthalpy of melting for 100% crystalline polymer. *ΔH*°_m_ for PLA is 93 J/g.

**Table 1:** Crystallinity of the PLA samples based on DSC measurements data

Sample	ΔHcc (J/g) 1st heating	ΔH°m (J/g) 1st heating	% Crystallinity 1st heating	Melting point (°C) 1st heating
PLA pellets	No peak	33.0	35.4	153.6
PLA film	0.5	2.3	1.9	152.3

### Enzymatic biodegradation

Enzymatic hydrolysis of PLA samples was performed following standard protocols (Ponjavic et al. 2017b, 2018) by using enzyme mix of alcalase 2.4 L FG (Novozymes, batch PLN05554), savinase (Novozymes, GHSFS-1-02-1) and 3 lipases (Serowar PL; Sigma, cat.no. 54327 and Sigma, cat.no. 52001). Enzyme mix contained 5 mg/mL of alcalase and savinase, and 1.5 mg/mL of each of lipases in 20 mM Tris- HCl buffer (pH 8.5) and was stored at − 20 °C.

Film samples of approximately 50 mg were placed in glass vials and 6 mL of 20 mM Tris- HCl buffer (pH 8.5) was added. The reaction started by addition of 1 mL of enzyme mix. Control reactions contained no enzyme mix. Samples were incubated at 42 °C at 150 rpm for 16 weeks. Weekly, aliquots (1 mL) were taken and stored at -20 °C for analysis, while at the same time 1 mL aliquots of enzyme mix were added.

### HPLC analysis of PLA hydrolysates

Samples were prepared for HPLC analysis by adding 50 µL of 6 M HCl to 1 mL of the hydrolysates, mixed using vortex and centrifuged for 10 min at 12 000 × rpm (Eppendorf Centrifuge 5417 R, Germany). The supernatants were filtered through 0.2 µm syringe filters directly into clean vials. For the analysis an UltiMate 3000 HPLC (Thermo Fisher Scientific, USA) system equipped with a Eurospher II 100-3 C18A 150 × 4.6 mm (Knauer, Germany) column was used. The separation was performed under isocratic conditions at a flow rate of 1 mL/min and the mobile phase consisted of 95% (v/v) 20 mM NH_4_H_2_PO_4_ in ultrapure water (adjusted to pH 2 with H_3_PO_4_) and 5% (v/v) acetonitrile. The degradation products were detected at 210 nm according to De Baere et al. (2013) and compared to standard lactic acid.

### Bacterial nanocellulose production

PLA hydrolysates from enzymatic biodegradation were used as substrate for nanocellulose production using *Komagataeibacter medellinensis* ID13488 (Jeremic et al. 2019). *Komagataeibacter medellinensis* strain transforms glucose to glucose-6-phosphate, glucose-1-phosphate, uridine diphosphate (UDP)-glucose, and finally to unbranched *β*-1,4-D-glucan i.e., bacterial nanocellulose, in the presence of glucokinase, phosphoglucomutase and uridine triphosphate (UTP)-glucose-1-phosphate uridylyltransferase, with the aid of cellulose synthases. Although glucose is considered the main source, all substrates that can be transformed to glucose are theoretically available for nanocellulose production (Wang et al. 2018). It is expected that during PLA degradation lactic acid is transformed to fructose through pyruvaldehyde and glyceraldehyde intermediers, and then isomerized to glucose, which is used by bacteria for nanocellulose production.

In order to prepare inoculum, *K. medellinensis* strain was cultivated in a standard Hestrin–Schramm medium pH 4.5 (HS) containing 20 g/L glucose, 5 g/L peptone, 5 g/L yeast extract, 2.5 g/L Na_2_HPO_4_and 1.15 g/L citric acid, under static conditions at 28 °C. Volume of 2 mL of PLA hydrolysates was poured to the glass tubes, pH was adjusted to 4.5 using concentrated HCl, and inoculum of 200 µL of bacterial culture was added (10% *v/v,* inoculum). All samples were incubated under static conditions at 28 °C for 7 days. After incubation, nanocellulose discs were treated with 5% KOH aqueous solution and extensively washed with distilled water until a pH of 7.0 was reached, air dried and weighted. Nanocellulose production was presented as g/L of medium obtained after 7 days.

## Results and discussion

### Degradation in compost

Biodegradation of control and pretreated PLA films in model compost showed small morphological changes in the shape and coloration of the samples treated for 24 weeks as can be seen in Figure S1.

Weight loss of only 0.2% and 0.6% after 10 and 24 weeks, respectively, was detected in the PLA samples pretreated using ultrasonic waves (US) with a frequency of 860 kHz. As already reported in our previous paper (Kalogirou et al. 2022) this is most probably due to the chemical interaction of the samples treated with 860 kHz leading to a shift of the C–O and C=O bonds to C–C bonds but also to changes of the PLA surface due to the strong oscillatory behavior of the cavitation bubbles at 860 kHz. Although the detected weight loss seems modest, it must be considered that promising results reported in the literature come from composting PLA under industrial conditions, at elevated temperatures (45–60 °C) (Kulikowska et al. 2020; Kalita et al. 2021a, 2021b), while in this study the composting was performed at 37 °C. Wilfred and coworkers showed similar weight loss of PLA films, 0.7%, after two-week incubation at 45 °C (Wilfred et al. 2018), while weight loss of up to 90% is characteristic for composting at close to 60 ºC (Kawashima et al. 2021; Boonmee et al. 2016). Currently, temperatures achieved in home composts are lower than ASTM/ISO test temperatures, so that compostable PLA in a home pile is unlikely to complete the process of biodegradation within the time limits. Recent study of PLA degradation in home compost (Solano et al. 2022) showed that even after 31 weeks of incubation at room temperature no biodegradation was observed. However, this study highlights the possibility of optimizing PLA pretreatment towards improved composting in mild, home composting conditions, thus leading to reduced greenhouse gas (GHG) emissions and better waste management globally. Until proper PLA composting is established, not only in composting plants, but in households as well, it is somewhat pointless to use PLA and other biodegradable plastic products, since they will not contribute to waste reduction drastically (Kawashima et al. 2021).

The total number of viable microorganisms, represented as CFU/g, after 10 weeks of degradation in model compost showed slight changes in number, within the error range, in all three tested growth media, while after 24 weeks a moderate increase in CFU/g was observed. This suggest that PLA degradation in model compost did not significantly affect the total number (Table S1) and visually noticeable diversity of present microorganisms (Figure S2), which is correlating with similar studies (Esan et al. 2019). The only significant difference is reduction of microorganism counts by two orders of magnitude from T0 to after 10 weeks incubation (LA). Reduction of one order of magnitude is due to the incubation of fresh compost sample for prolonged period in closed glass petri plates, and it is considered expected. The phenomenon of increase in the number of bacteria in compost after degradation is also observed in several other studies (Orhan et al. 2004; Gautam and Kaur 2013), indicating that plastic degradation products may stimulate the growth of certain bacterial species. Even though it is shown that PLA composting had no significant effect on the bacterial count, it is necessary to perform a detailed microbial community analysis to determine variations in bacterial composition.

### Characterization of the composted samples and comparison with reference samples

**Fig. 1:**
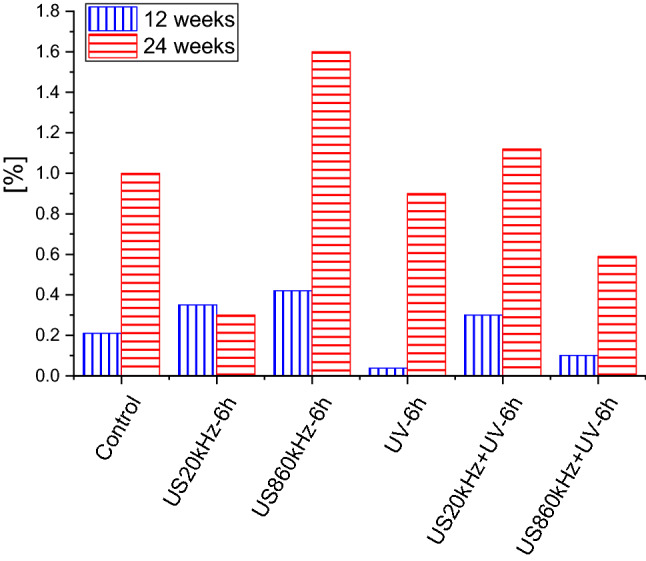
Mass loss of the pre-treated PLA samples after exposition to model compost (MC) for 12 and 24 weeks

The pre-treated PLA samples have been weighed after exposition for 12 and 24 weeks to model compost (MC) and it has been found that depending on exposition time and pre-treatment method different degradation percentages result as summarized in Fig. 1.

Taking a closer look on the XPS (x-ray photoelectron spectroscopy) results of the pretreated PLA samples (Table S2) it is interesting to point out that the samples with the highest degradation rate (except the one at 12 weeks–20 kHz and 6 h treatment) contain nitrogen after the long compost contact time of 24 weeks (Kalogirou et al. 2022). That indicates the chemical involvement of nitrogen from ambient air in the degradation mechanism of pre-treated PLA. As the blind sample also contains nitrogen after 24 weeks the nitrogen involvement seems to be independent of the pre-treatment method.

**Fig. 2:**
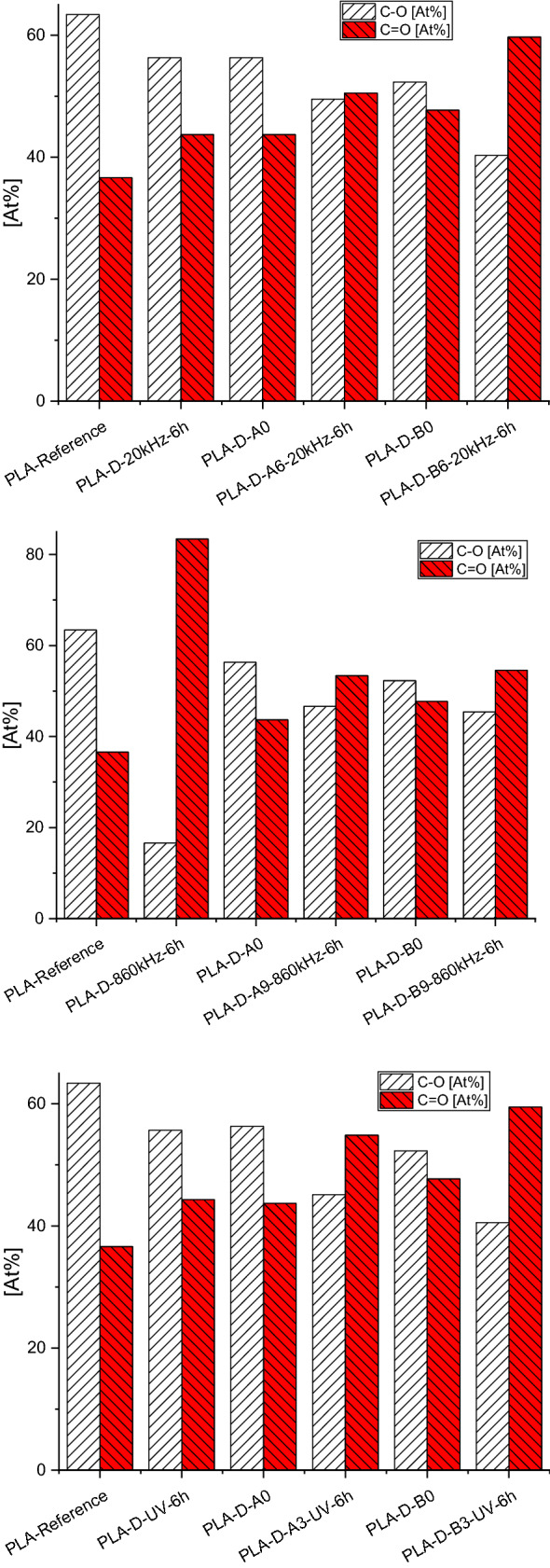
Development of the oxygen bond to the carbon of (top) 20 kHz ultrasounds, (middle) 860 kHz ultrasounds and (bottom) UV treated PLA samples with exposure time to model compost

From Fig. 2 it is obvious that during the compost exposition the degradation occurs over a mechanism that converts single bonded PLA oxygen to double bonded carboxylic oxygen following a mechanism as proposed by Song et al. (2016). Similarly, the strong increase in –CH_3_ percentage (Table S2) can be explained by the same mechanism, as –CH_3_ is obtained from both proposed reaction pathways.

**Fig. 3:**
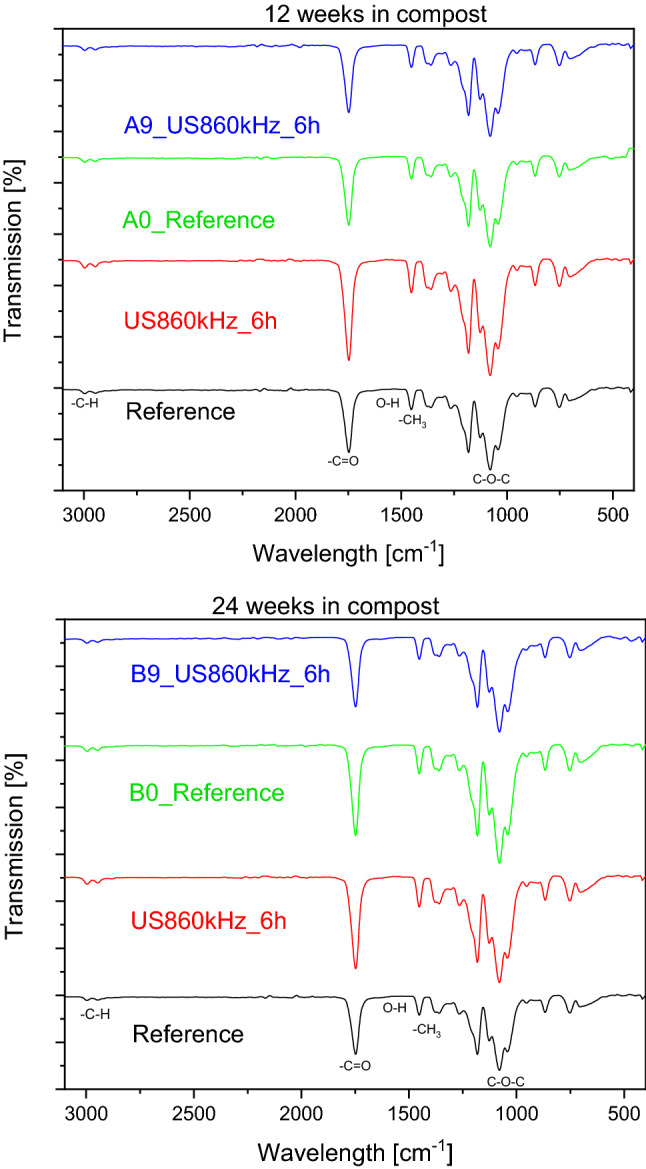
IR spectra of the PLA samples treated using HFUS at 860 kHz before and after composting. (top) after 12 weeks in model compost (bottom) after 24 weeks in model compost

It has been reported (Mofokeng et al. 2012), that in IR spectra the existence of the groups C=O and CH_3_ results in the appearance of peaks at wavelength 1747 cm^−1^ and 1455 cm^−1^ respectively. From the IR spectrum we get the information that the larger the peak, the lower the amount of the respective group in the sample as it is given in transmission. Based on that, the behavior found by XPS is confirmed also by IR-spectra of the samples degraded in compost for 12 and 24 weeks (Fig. 3).

### Enzymatic degradation of PLA films

Based on the vast literature data on enzymatic degradation of PLA (Zaaba and Jaafar 2020) and the fact that serine proteases as the main member of PLA-degrading proteases, but also some alkaline proteases, lipases and cutinases showed the activity, we have included commercial serine endo-peptidase active on variety of pH and temperatures as well as three lipases in the enzyme mix and exposed PLA films to their activity over prolonged period of time (up to 16-weeks) at 42 °C whereby high load of enzymes per amount of material was applied (15% w/w) and fresh aliquot of enzyme mix was supplied regularly.

**Fig. 4:**
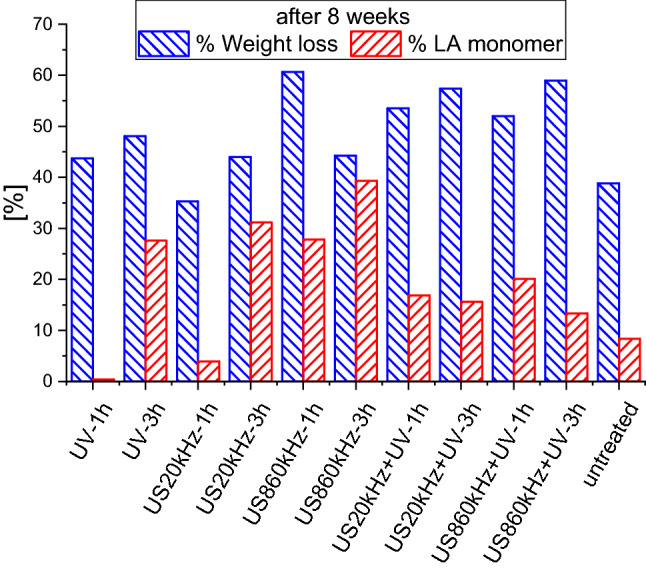
Weight loss in [%] of selected PLA samples after 8 weeks of enzymatic degradation and corresponding lactic acid (LA) monomer in [%] of weight loss

In Fig. 4 the weight losses of selected PLA samples after 8 weeks of enzymatic degradation are presented along with the amount of produced lactic acid (monomer) in [%] of the corresponding weight loss for each pre-treatment method. It is obvious that ultrasounds at 860 kHz is the most effective method for activating the degradation of PLA and its combination with UV for 3 h to be the most effective pretreatment method. For the degradation of PLA in compost ultrasounds at 860 kHz exhibit also the highest degradation (Fig. 1).

After 8 weeks the conversion rate to the lactic acid monomer is highest in the 860 kHz pre-treated samples for 3 h. Ultrasounds have the most positive impact on the production of the pure lactic acid monomer as it leads to a conversion of the pre-treated PLA to lactic acid monomer of 40%. High frequency US shows high degradation rates and the conversion to monomer is increasing as the pre-treatment time increases from 1 to 3 h.

**Fig. 5:**
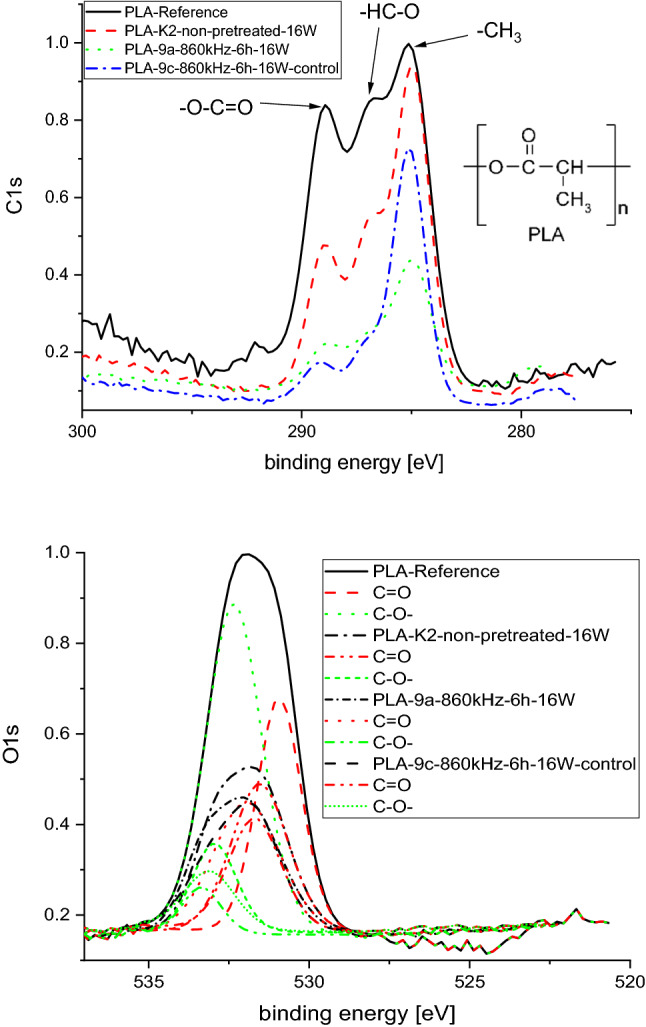
(Top) Normalized XPS C1s signal showing the influence of the enzymatic degradation on the surface chemistry of PLA, with a formula for PLA showing the different carbon bond types. (bottom) Normalized XPS O1s signal with deconvoluted C–O and C=O peaks

XPS results (Fig. 5) of the enzymatic degraded samples (here exemplarily the sample pre-treated with high frequency ultrasounds (HFUS) at 860 kHz for 6 h, which exhibited the highest degradation rate after 16 weeks) show that oxygen is used in the degradation reaction as it is diminished in the pre-treated sample.

IR spectra (Figure S1) confirm the existence of increased C=O concentration after 6 h at 860 kHz ultrasounds relative to C–O. This trend is pertained also after the enzymatic treatment of the samples as can be seen in Figure S4 in, meaning that oxygen is most probably needed only for the attachment of the enzymes and not involved in the degradation reaction. If oxygen is involved in the degradation reaction, then the high oxygen concentration in the degraded samples could be only explained if they have been continuously oxidized by the enzyme.

**Fig. 6:**
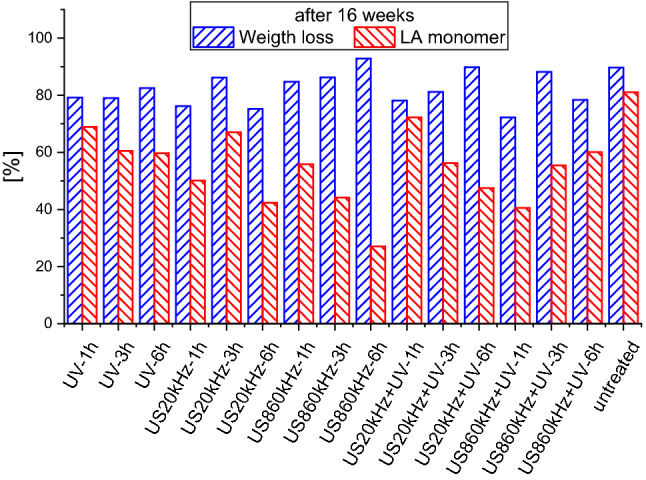
Weight loss in [%] of selected PLA samples after 16 weeks of enzymatic degradation and corresponding lactic acid (LA) monomer in [%] of weight loss

Considering the enzymatic degradation of all samples treated for 1 and 3 h after 8 and 16 weeks it was surprising that the untreated sample after 16 weeks exhibits an outstanding performance and competes with the pretreated samples (Fig. 6). It exhibits a similar performance as the samples treated with low frequency ultrasounds for 3 h. This is surprising at first glance, as ultrasounds at 20 kHz have a high impact on the surface morphology because of the high power of the US at this frequency, which leads to large cavitation bubbles. The explanation for this behavior lies on the fact, that the collapse of the cavities is so violent that after 3 h the surface is eroded and thus (at least partly) like the fresh one, as already reported (Sourkouni et al. 2021). Further these results indicate that once the right enzyme cocktails are found the degradation of PLA and most probably of any other plastic are possible without or with less polymer surface activation.

**Fig. 7:**
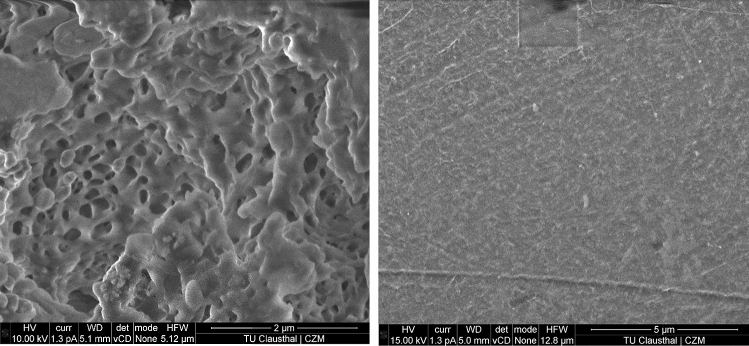
SEM micrographs of the sample (left) pre-treated for 6 h at 860 kHz + UV after enzymatic degradation for 16 weeks and (right) pristine PLA sample

In Fig. 7 SEM micrographs are showing the surface changes and the bulk porosity induced to the samples (here the sample which was pre-treated at 860 kHz + UV for 6 h). The porosity is indirectly ascertained during drop contour measurements, where the drop flowed through the sample.

It has been recently reported that cutinases and esterases can degrade most plastics that are composed of polyester constituents. On the other hand, serine proteases such as proteinase K, trypsin, elastase, and chymotrypsin are able to depolymerise PLA into its dimers and monomers by a two-step hydrolysis reaction (Qi et al. 2017; Gan and Zhang 2019).

Proteinase K of fungal origin is the first enzyme ever reported to degrade PLA in 1981, with a temperature optimum of 50 °C (Williams 1981). Since then, none of the commercially available acid, neutral and alkaline proteases showed higher efficiency in PLA degradation in comparison to Proteinase K (Lim et al. 2005**).** Among 56 commercial proteases with industrial application, Savinase showed to be the second best in PLA degradation yet achieved only 50% of Proteinase K activity at 50 °C (Oda et al. 2000b**).** Generally, alkaline proteases showed to be the best, some neutral proteases showed to be active and acid proteases showed to be ineffective as PLA depolymerases. In 2005 cutinase from *Cryptococcus* sp. strain S-2 (CLE) with high-molecular-weight PLLA degradation 500 times more efficient in comparison to Proteinase K at 30 °C was discovered (Masaki et al. 2005).pH and temperature optimum for the majority of PLA degrading enzymes show that enzyme catalysis preferentially occurs at elevated temperatures above 37 °C and up to 70 °C in alkaline conditions, therefore activities of these enzymes are compatible with PLA melting and abiotic autocatalytic degradation processes (Sukkhum and Kitpreechavanich 2011). Comparative study of PLA degradation by *Candida cylindracea* lipase, pig liver esterase and *Bacillus licheniformis* alcalase at optimum conditions for each enzyme. Results showed that alcalase is the most potent enzyme that degraded PLA sample to microplastics, resulting in 25% weight loss after 21 days at pH 9.5 and 60 °C unlike in the case of lipase and esterase (1.4% weight loss) at pH 8 and 40 °C (Lee et al. 2014). Complete PLA degradation by Lipase PL was achieved after 20 days at 55 °C, pH 8.5 while the same sample remained intact at 37 °C, pH 7 throughout 100 days of incubation (Hoshino and Isono 2002). Poly (DL-lactide) (average molar mass Mw 1.0–1.8 × 10^4^) powder was degraded by almost 40% by 50 µg of RPA1511 (protein from *Rhodopseudomonas palustris*), and 90% by 50 µg of ABO2449 (protein from *Alcanivorax borkumensis*) upon 0.1% surfactant addition after 36 h of incubation at 35 °C and pH 8.0. Both enzymes were inactive towards either form of enantiopure PLA (Hajighasemi et al. 2016). Proteinase K and CLE decreased the weight of PLLA and PDLA films (15 mm × 5 mm) after 4 days at 37 °C by 7% and 14%, respectively (Kawai et al. 2011). The first non-commercial protease-type PLA depolymerase was purified from *Amycolatopsis sp*. strains K104-1 and it degraded over 90% of PLA film at 37 °C and pH 8.6 after 48 h with extensive pH control achieved through dialysis of reaction to prevent enzyme inactivation by pH drop throughout reaction (Nakamura et al. 2001). Xu et al. provided an overview of enzymes, PLA substrates, degradation conditions and percent degradation reported in the literature from 1997 to 2016 (Xu et al. 2022). Report on PLA film weight reduction by 26% was achieved by purified lipase from *Sphingobacterium sp.* strain S2 at 37 °C and pH 8 after 72 h (Satti et al. 2019).

Based on all these data, we made a cocktail of commercially available enzymes including Alcalase FG, Savinase and 3 lipases including Lipase PL and optimized enzymatic process to achieve 90% weight-loss at 42 °C and at mild alkaline conditions (pH 8.5) over 16 weeks (Figs. 4, 5, 6, 7). The reported weight loss was achieved only in a combination of pretreatment and enzymatic degradation, not pretreatment alone. There is room for further optimization of the bioprocess, but these results are a huge step in biotechnological enhancement of the PLA depolymerisation and subsequent valorization of the hydrolysis products.

#### Nanocellulose production from enzymatic PLA hydrolysate

**Fig. 8:**
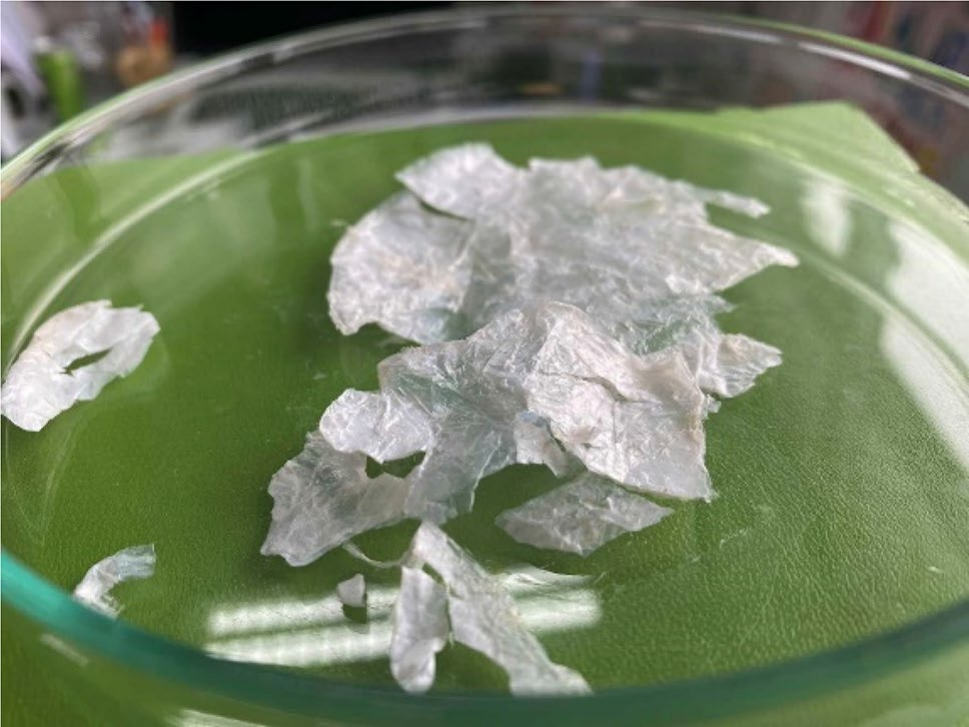
Produced cellulose from PLA degradation liquid residues

Acidified enzymatic PLA hydrolysate successfully supported growth and nanocellulose production by *K. medellinensis* within 3 days of incubation (Figure S4). After 7 days of incubation, dry nanocellulose was weighted and the results are presented in Table 2 and the dried nanocellulose in Fig. 8. Obtained nanocellulose yields were comparable with nanocellulose obtained from standard HS medium supplemented with 2% glucose. Slightly higher nanocellulose production was supported by hydrolysates of PLA pre-treated with US860kHz and US860kHz + UV during 6 h (Table 1).

**Table 2:** Nanocellulose production in g/L liquid residues from the enzymatic degradation of PLA

Sample	Nanocellulose (dw, g/L)
UV-1 h	3.4
UV-3 h	3.4
UV-6 h	3.9
US20kHz-1 h	3.6
US20kHz-3 h	3.8
US20kHz-6 h	4.0
US860kHz-1 h	2.9
US860kHz-3 h	4.2
US860kHz-6 h	4.3
US20kHz + UV-1 h	2.5
US20kHz + UV-3 h	2.7
US20kHz + UV-6 h	1.8
US860kHz + UV-1 h	3.9
US860kHz + UV-3 h	3.8
US860kHz + UV-6 h	4.3
non pretreated PLA	4.0
Control (glucose)^*^	5.8

*Nanocellulose produced in HS medium supplemented with 2% (w/w) glucose

The results in Table 1 indicate that the monomer amount depends on the pre-treatment method and the treatment time. For example, despite the high nanocellulose yield using 20 kHz ultrasounds and UV (Fig. 4), their combination most probably degrades also the monomer, and it is no more available for the nanocellulose production leading to low yields (1.8 g/L with US20kHz + UV-6 h).

Previously, increased bacterial nanocellulose production was obtained in the presence of lactic acid by *Acetobacter* sp. V6 (Jung et al. 2010).

It has been shown in the recent literature that the nanocellulose produced by bacteria is nano-dimensional (Ludwicka et al. 2020; Sharma et al. 2019; Trache et al. 2020). There are generally three types of nanocelluloses: cellulose nanocrystals, cellulose nanofibrils and bacterial cellulose. During nanocellulose production by bacteria, numerous polymerized *β*-1,4 glucan chains are secreted outside the bacterial cell through a linear array of 3.5 nm pores on the outer membrane. Once out, the *β*-1,4 glucan chains are assembled in a precise and hierarchical process. First, sub-fibrils form, consisting of 10 to 15 chains of nascent glucans. Then, these sub-fibrils assemble and crystallize to form fibrils, which then combine to form a cellulose nanofiber and microfiber comprised of about 1000 individual glucan chains (30–100 nm diameter).

## Conclusions

This study aimed to assess the effectiveness of different advanced oxidation processes (AOPs) for PLA pre-treatment on improving its enzymatic degradability and compostability under mesophilic conditions. Since AOPs modify the surface of PLA towards enhancing bacterial docking, the action of bacterial enzymes and degradation itself is facilitated. Obtained results corroborated assumption, with excellent PLA degradation—up to 90% weight loss—achieved using US/UV pre-treatment and enzyme hydrolysis. The established experimental setup will enable study extension on other pre-treatments and their impact on the PLA biodegradability. Degradation of pre-treated PLA samples in model compost under mild conditions was also proven in this study, emphasizing the possibility of home composting of PLA materials in the future.

In this respect a commercial enzyme mix containing serine endo-peptidases and lipases was found to be highly efficient in PLA hydrolysis. For the first time, we report valorization of PLA into bacterial nanocellulose after enzymatic hydrolysis of the samples. It has been found that the monomer amount depends on the pre-treatment method and the treatment time. Depending on the pre-treatment method between 1.8 and 4.3 g/L nanocellulose have been produced.

In this study for the first-time products of PLA degradation were valorized into bacterial nanocellulose, proving thus the possibility of a circular economy concept for PLA. Future studies will be focused on bioprocess optimization towards higher nanocellulose yields and characterization of such obtained material.

## Supplementary Information

Below is the link to the electronic supplementary material.

Supplementary file1 (DOCX 725 KB)

## Data Availability

Most of the raw data can be found in the publication itself (XPS, IR spectra) and in the supplemental information file of the present publication. Additional raw data cannot be published at present as it is an onoing European Project. Nevertheless data can be made available to the community upon request.
