# Supplementary material for: Study of PLA pre-treatment, enzymatic and model-compost degradation, and valorization of degradation products to bacterial nanocellulose
Source: World J Microbiol Biotechnol. 2023 Apr 17;39(6):161. doi: 10.1007/s11274-023-03605-4 (PMC10110681; doi:10.1007/s11274-023-03605-4)
Supplement: Supplementary file 1 — Supplementary file1 (DOCX 725 KB) [file 11274_2023_3605_MOESM1_ESM.docx]

**Supporting information**

**Study of PLA pre-treatment, enzymatic and model-compost degradation, and valorization of degradation products to bacterial nanocellulose**

Georgia Sourkouni^a,^*, Sanja Jeremić^b^, Charalampia Kalogirou^a,c^, Oliver Höfft^d^, Marija Nenadovic^b^, Vukasin Jankovic^b^, Divya Rajasekaran^e^, Pavlos Pandis^c^, Ramesh Padamati^e^, Jasmina Nikodinovic-Runic^b^, and Christos Argirusis^c,a^

^a^ Clausthal Centre for Materials Technology (CZM), Clausthal University of Technology, Leibnizstr. 9, 38678 Clausthal-Zellerfeld, Germany

^b^ Institute of Molecular Genetics and Genetic Engineering (IMGGE), University of Belgrade (UB), Vojvode Stepe 444a, 11042 Belgrade 152, Serbia

^c^ School of Chemical Engineering, National Technical University of Athens, 9 Heroon Polytechneiou St., Zografou Campus, 15773 Athens, Greece

^d^ Institute for Electrochemistry, Clausthal University of Technology, 38678 Clausthal-Zellerfeld, Germany

^e^ School of Chemistry, Trinity College Dublin, College Green, Dublin 2, Ireland

[* Corresponding author: cogsa@tu-clausthal.de](mailto:*cogsa@tu-clausthal.de)


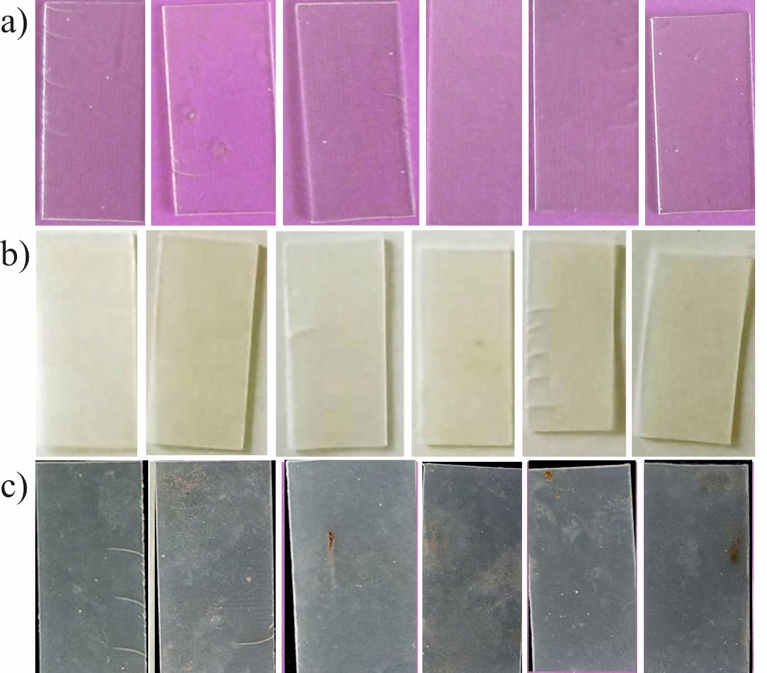


Figure S1: Morphological appearance of PLA films: a) before degradation in compost, b) after 10 weeks of composting, c) after 24 weeks of composting. The order is as follows from left to right: control PLA, PLA UV 6h, PLA US 20kHz, PLA US 860kHz, PLA UV 6h/US 20kHz, PLA UV 6h/US 860kHz.


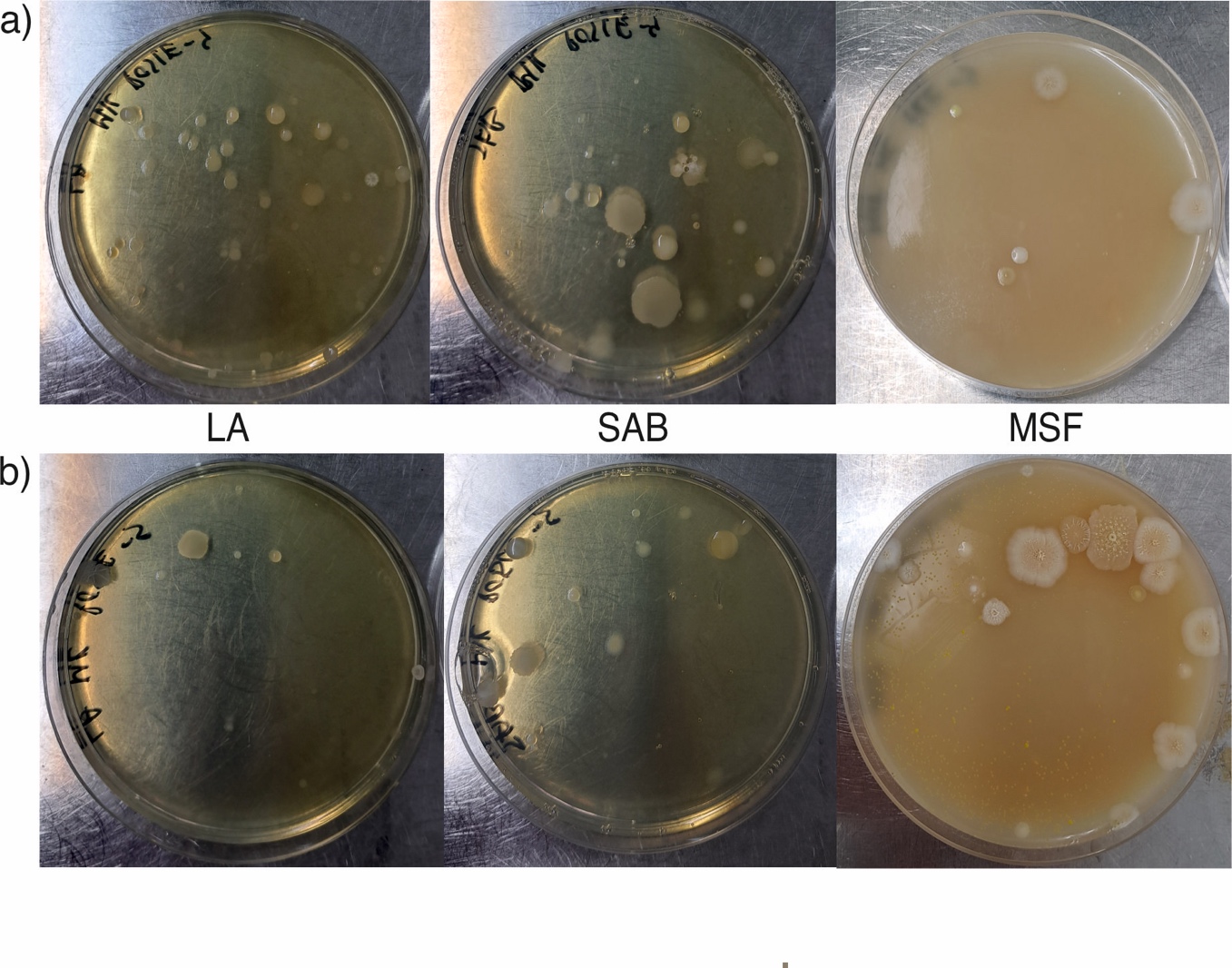


Figure S2: Morphologically observable microbial diversity on LA, SAB and MSF solid medium plates: a) before and b) after 24 weeks of degradation in model compost.

|  |
| --- |
| Figure S3: IR spectra of reference and blind samples with samples treated with HFUS at 860 kHz. |


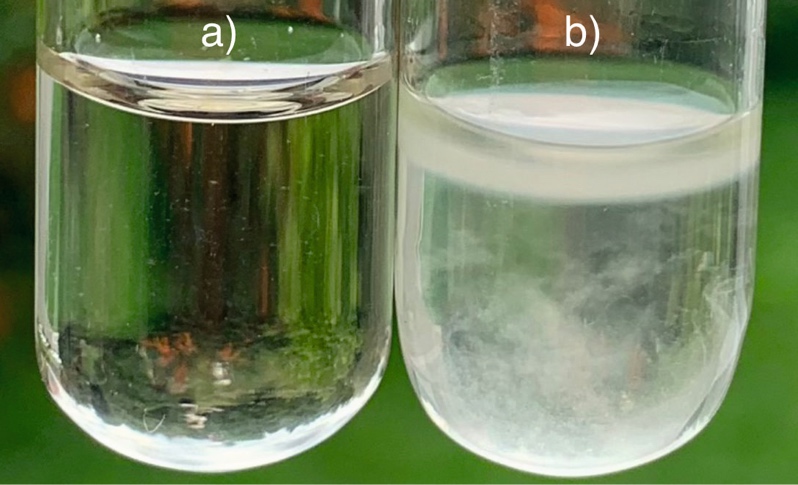


Figure S4: Bacterial nanocellulose production from PLA hydrolysates after three days of incubation: a) control and b) hydrolysate obtained after enzymatic degradation.

Table S1: Total number of viable microorganisms, represented as CFU/g, after 10 and 24 weeks of degradation in model compost.

|  | **CFU/g compost** | | |
| --- | --- | --- | --- |
| **growth media** | **T0** | **after 10 weeks** | **after 24 weeks** |
| MSF | 6.4 x 10^7^ | 6.7 x 10^6^ | 2.0 x 10^6^ |
| LA | 1.1x 10^8^ | 3.8 x 10^6^ | 1.6 x 10^6^ |
| SAB | 6.6 x 10^7^ | 2.6 x 10^6^ | 1.0 x 10^6^ |

Table S2: XPS results of the pretreated and composted PLA samples for A=12 weeks and B=24 weeks (A and B in the sample name). C1s stands for the photoelectrons from the carbon electronic state associated with sp2 and sp3 hybridized carbon. O1s stands for the photoelectrons from the 1s electronic state of oxygen.

|  | C1s [At%] | O1s [At%] | N1s [At%] | CH3- [At%] | CH-O- [At%] | C=O [At%] | C-O [At%] | C=O [At%] |
| --- | --- | --- | --- | --- | --- | --- | --- | --- |
| PLA-Reference | 59.4 | 40.56 |  | 40.24 | 31.58 | 28.18 | 63.38 | 36.62 |
| PLA-D-20kHz-6h | 60.93 | 39.07 |  | 51.37 | 23.57 | 25.06 | 56.31 | 43.69 |
| PLA-D-860kHz-6h | 56.23 | 43.77 |  | 80.91 | 10.47 | 8.62 | 16.59 | 83.41 |
| PLA-D-UV-6h | 71.47 | 28.53 |  | 67.48 | 16.65 | 15.87 | 55.68 | 44.32 |
| PLA-D-20kHz+UV-6h | 67.61 | 31.57 | 0.82 | 61.2 | 18.49 | 20.3 | 49.68 | 50.32 |
| PLA-D-860kHz+UV-6h | 59.12 | 40.88 |  | 51.21 | 21.44 | 27.35 | 53.41 | 46.59 |
| PLA-D-A0 | 60.28 | 39.72 |  | 47.17 | 27.02 | 27.81 | 56.31 | 43.69 |
| PLA-D-A6-20kHz-6h | 66.2 | 32.92 | 0.88 | 63.49 | 16.9 | 19.61 | 49.51 | 50.49 |
| PLA-D-A9-860kHz-6h | 66.33 | 33.67 |  | 64.12 | 16.82 | 19.06 | 46.63 | 53.37 |
| PLA-D-A12-20kHz+UV-6h | 59.93 | 39.45 | 0.62 | 49.32 | 26.78 | 23.9 | 30.96 | 69.04 |
| PLA-D-A15-860kHz+UV-6h | 63.49 | 35.38 | 1.13 | 56.87 | 22.61 | 20.52 | 41.86 | 58.14 |
| PLA-D-A3-UV-6h | 61.65 | 38.35 |  | 49.13 | 27.12 | 23.75 | 45.12 | 54.88 |
| PLA-D-B0 | 61.4 | 38.06 | 0.54 | 56.79 | 20.86 | 22.35 | 52.3 | 47.7 |
| PLA-D-B6-20kHz-6h | 59.62 | 38.63 | 1.74 | 48.89 | 23.96 | 27.16 | 40.3 | 59.7 |
| PLA-D-B9-860kHz-6h | 53.22 | 46.06 | 0.72 | 43.13 | 31.02 | 25.85 | 45.43 | 54.57 |
| PLA-D-B3-UV-6h | 54.83 | 44.09 | 1.08 | 46.36 | 29.51 | 24.13 | 40.55 | 59.45 |
| PLA-D-B12-20kHz+UV-6h | 59.94 | 39.51 | 0.55 | 55.06 | 26.72 | 18.22 | 39.47 | 60.53 |
| PLA-D-B15-860kHz+UV-6h | 61.8 | 36.73 | 1.47 | 56.84 | 21.34 | 21.82 | 36.55 | 63.45 |
